# A Portable Device for LAMP Based Detection of SARS-CoV-2

**DOI:** 10.3390/mi12101151

**Published:** 2021-09-24

**Authors:** Kamalalayam Rajan Sreejith, Muhammad Umer, Larissa Dirr, Benjamin Bailly, Patrice Guillon, Mark von Itzstein, Narshone Soda, Surasak Kasetsirikul, Muhammad J. A. Shiddiky, Nam-Trung Nguyen

**Affiliations:** 1 Queensland Micro- and Nanotechnology Centre, Griffith University, 170 Kessels Road, Nathan, QLD 4111, Australia; m.umer@griffith.edu.au (M.U.); narshone.soda@griffithuni.edu.au (N.S.); surasak.kasetsirikul@griffithuni.edu.au (S.K.); m.shiddiky@griffith.edu.au (M.J.A.S.); 2 Institute for Glycomics, Gold Coast Campus, Griffith University, 1 Parklands Drive, Southport, QLD 4222, Australia; l.dirr@griffith.edu.au (L.D.); b.bailly@griffith.edu.au (B.B.); p.guillon@griffith.edu.au (P.G.); m.vonitzstein@griffith.edu.au (M.v.I.); 3 School of Environment and Science, Nathan Campus, Griffith University, 170 Kessels Road, Brisbane, QLD 4111, Australia; 4 School of Engineering and Built Environment, Nathan Campus, Griffith University, 170 Kessels Road, Nathan, QLD 4111, Australia

**Keywords:** severe acute respiratory syndrome-coronavirus 2 (SARS-CoV-2), loop-mediated isothermal amplification, portable device

## Abstract

This paper reports the design, development, and testing of a novel, yet simple and low-cost portable device for the rapid detection of SARS-CoV-2. The device performs loop mediated isothermal amplification (LAMP) and provides visually distinguishable images of the fluorescence emitted from the samples. The device utilises an aluminium block embedded with a cartridge heater for isothermal heating of the sample and a single-board computer and camera for fluorescence detection. The device demonstrates promising results within 20 min using clinically relevant starting concentrations of the synthetic template. Time-to-signal data for this device are considerably lower compared to standard quantitative Polymerase Chain Reaction(qPCR) machine (~10–20 min vs. >38 min) for 1 × 10^2^ starting template copy number. The device in its fully optimized and characterized state can potentially be used as simple to operate, rapid, sensitive, and inexpensive platform for population screening as well as point-of-need severe acute respiratory syndrome-coronavirus 2 (SARS-CoV-2) detection and patient management.

## 1. Introduction

The ongoing severe acute respiratory syndrome-coronavirus 2 (SARS-CoV-2) pandemic has so far, as of 18 July 2021, caused around 190 million confirmed cases of infection and more than 4 million deaths worldwide [1]. Though many vaccines have been proved to be effective against the pandemic, the importance of non-pharmaceutical interventions such as physical distancing, contact tracing, and quarantine are still effectively used to control the spread of the disease [2]. However, experts have emphasized that social distancing measures cannot be effective without extensive testing for SARS-CoV-2. Around 80% of infected persons show mild or no symptoms at all, thus majority of the infections go undocumented. Mathematical modelling of spatiotemporal dynamics of SARS-CoV-2 spread have shown that undocumented cases are the source of infection for a large majority (79%) of documented cases, thus emphasizing the need for mass testing [3].

Quantitative reverse transcriptase polymerase chain reaction (qRT-PCR) is the current gold standard diagnostic test for SARS-CoV-2. Although the qRT-PCR method is well tested and assays for newly identified organisms can be developed within a very short period of time, it has several drawbacks. The test is relatively expensive and can only be carried out in dedicated centralized diagnostic laboratories. The test itself takes more than an hour to complete, however as the samples need to be shipped to distant laboratories, actual turnaround time may be more than 24 h [4]. In a rapidly spreading pandemic, particularly in the context of resource limited regions, reliance on expensive centralized testing may prove to be a bottleneck in implementation of control measures and clinical decision making. Therefore, there is an urgent need to develop point-of-care (POC) testing devices. Earlier this year, the WHO expert group identified the development of rapid POC tests for SARS-CoV-2 as the first of eight research priorities [5]. Consequently, several excellent new methods have been reported for rapid and POC di-agnostics of SARS-CoV-2. Some of the prominent POC SARS-CoV-2 detection methods include isothermal amplification methods like loop mediated isothermal amplification (LAMP) and Recombinase Polymerase Amplification (RPA) combined with Clustered Regularly Interspaced Short Palindromic Repeats (CRISPR) based viral RNA detection, electrochemical and plasmonic biosensors, as well as POC immunoassays especially lateral-flow assays. These emerging diagnostic platforms can prove to be game-changers in near future especially because as they can be potentially developed for rapid at-home testing [6].

Single temperature nucleic acid amplification methods such as loop-mediated isothermal amplification (LAMP) have emerged as simple alternatives to qRT-PCR. The LAMP method is highly specific, takes less than an hour to complete, is less affected by non-target templates and well-known PCR inhibitors present in biological samples, can be implemented even without DNA extraction, and lastly can directly amplify RNA templates without cDNA conversion [7] and complex sample handling procedures [8]. Fluorescence based real time monitoring of LAMP reaction is by far considered most sensitive compared to other methods [7]. However, detection usually relies on the fluorescence sensors integrated in conventional benchtop thermocyclers. In recent years, efforts have been made to develop instruments with dedicated fluorescence readout for isothermal amplification (e.g., Genie^®^ II by OptiGene). Similarly, the Lucira COVID-19 All-In-One Test Kit is an RT-LAMP-based platform that has recently been given FDA EUA and can be used for at-home testing of SARS-CoV-2 by people over 14 years of age [9]. However, these instruments are still relatively expensive because of the complex design. A simple, inexpensive, robust, and portable fluorescence detection enabled LAMP device can immensely strengthen the battle against SARS-CoV-2. Such a field deployable device will obviate the need for sophisticated sample storage and shipment chain thus reducing the testing cost and time gap between sample collection and delivery of results. Using LAMP for an RNA virus like SARS-CoV-2 can further reduce the cost and analysis time by eliminating the need for RNA isolation and cDNA conversion steps. Additionally, many on-chip LAMP based diagnosis and pathogen detection were reported recently [10,11,12,13,14,15,16], which could provide better sample handling platform for LAMP based disease diagnosis and pathogen detection. Taken together, these features make a LAMP based portable/handheld device an excellent candidate for mass screening and point-of-care patient management in our time of widespread pandemic. Herein we report a novel, low cost and portable device capable of performing LAMP reaction and sensitive monitoring of fluorescence in real time. Using this device, we demonstrate the proof of concept for detection of clinically relevant concentrations of SARS-CoV-2 specific sequences.

## 2. Materials and Methods

### 2.1. Culture of SARS-CoV-2 and Viral RNA Extraction

All experiments involving the culture and handling of SARS-CoV-2 were conducted in a certified physical containment level 3 (PC3) facility at the Institute for Glycomics, Griffith University. SARS-CoV-2 strain SARS-CoV-2-CoV-2/Australia/QLD02/2020 (GISAID ID: EPI_ISL_407896) was obtained from the Forensic and Scientific Services Unit of Queensland Health, Australia. It was propagated in Vero E6 cells in DMEM supplemented with penicillin/streptomycin and 2% fetal bovine serum, in a humidified atmosphere of 5% CO_2_ at 37 °C. Infection supernatants were clarified by centrifugation for 15 min at 4000× *g*, homogenised, aliquoted and stored at −80 °C. Virus titers were determined by focus forming assays and expressed as virus plaque forming units (pfu) per mL, as previously described [17].

The SARS-CoV-2 viral RNA (vRNA) was extracted from 750 µL of infection supernatant containing 1.4 × 10^6^ pfu/mL, using a NucleoSpin Virus Mini kit (MACHEREY-NAGEL, Dueren, Germany). vRNA was resuspended in ultrapure H_2_O and RNA extraction yields, here 190 ng/µL, determined using a NanoDrop spectrophotometer (Thermo Fisher Scientific, Waltham, MA, USA).

### 2.2. Design and Experimental Conditions for SARS-CoV-2 Specific LAMP Assay

A LAMP assay targeting 225 bp long region spanning nucleotide positions 15, 355 to 15, 579 of SARS-CoV-2 NCBI reference sequence (NC_045512.2) within the RdRp gene of viral genome was designed using open access online LAMP primer design software Primer Explorer V5 (Table 1). Viral RNA was extracted from SARS-CoV-2 grown in cell culture. Three different known starting concentrations/copy numbers (C. Ns) of SARS-CoV-2 genome, 1 × 10^2^, 1 × 10^4^, and 1 × 10^6^, were used as template in LAMP reactions. A no-template control (NTC) reaction where nuclease free water was added in place of template was also carried out. SARS-CoV and MERS-CoV control reactions used genomic sequences from bat SARS like coronavirus (GenBank: MG772933.1) and Middle East respiratory syndrome-related coronavirus (GenBank: MK796425.1), respectively, and were obtained from IDT (Ref# 103374059 & 103374058 respectively). Target sequence was isothermally amplified using WarmStart^®^ LAMP Kit (DNA & RNA), (Cat *#* E1700S, New England Biolabs, Notting Hill, Australia) in a 25 µL reaction as per manufacturer’s instruction. The reaction mix consisted of 12.5 µL of WarmStart LAMP 2 × Master Mix, 0.5 µL of 50 × fluorescent dye, and 2.5 µL of 10× primer mix (final concentrations: FIP/BIP 1.6 µM, F3/B3 0.2 µM, LF/LB 0.4 µM). A volume of 1 µL of target of known copy number was used and the reaction was made up to 25 µL by adding 8.5 µL nuclease-free distilled water (Cat # 10977015, ThermoFisher Australia).

To compare the efficiency of our device, real time monitoring of change in fluorescence levels in LAMP reactions was carried out in parallel on CFX96 Touch Real-Time PCR Detection System-Bio-Rad. Samples were incubated on 65 °C for 30 min and fluorescence signal was recorded in FAM channel after every minute. The reaction was stopped by denaturing *Bst* 2.0 and RTx enzymes at 85 °C for 5 min. The melt curve analysis of amplified products was carried out by heating for 5 s each between 65 °C and 95 °C at 0.5 °C increments. Data collection was enabled at each increment of the temperature. Similar to the reaction set up on our device, LAMP reaction was carried out for three different starting template C. Ns as well as NTC, and MERS and SARS controls. All LAMP reactions on Bio-Rad qPCR platform were carried out in duplicate.

### 2.3. Device Design

The device consists of two subsections, the thermal control subsystem and the fluorescence monitoring subsystem. The thermal control subsystem is responsible to maintain the temperature required for loop-mediated isothermal amplification. A custom-designed aluminum block (40 × 30 × 10 mm^3^) with a cartridge heater (MG-1007, Makergear) embedded in it acts as the heater platform for sample holding. A custom-designed through-hole was made in the aluminum block so that the conventional PCR tube with the sample can be tightly inserted into it. The design of the sample placing hole is in such a way that there is an efficient heat transfer between the aluminum block and sample. The through-hole also helps for efficient fluorescence capturing on successful LAMP of the sample. An LM-35 temperature sensor was fixed to the aluminum block using Stars 922 heat conductive adhesive. The temperature of the aluminum block was fed back to a custom developed Atmega-328p microcontroller board (Arduino) programmed with a PID temperature controller algorithm. The power to the cartridge heater was controlled by the microcontroller board through a solid-state relay (KSJ30D100-L, Kudom, Jaycar, Australia) to regulate the temperature of the aluminium block to the set point temperature (65 °C).

**Figure 1:**
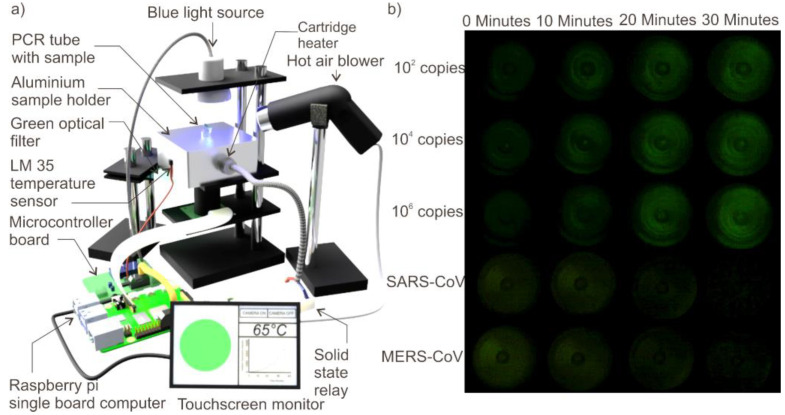
(**a**) Schematic of the experimental setup. (**b**) Photographs of fluorescence emitted from various samples upon performing the loop mediated isothermal amplification (LAMP).

Fluorescence monitoring subsystem consists of a raspberry pi B+ single-board computer connected with a 5MP Camera (B0032, Arducam), 7-inch touchscreen monitor (CE04459, Core electronics) and a blue light source. The blue light source (450–490 nm) was custom built by circularly arranging 30 blue LEDs (ZD0180, Jaycar, Australia). A graphical user interface (GUI) was developed using Python and installed in the single board computer to control the blue light source, camera and the monitor. The camera and the blue light source were arranged vertically and directed opposite to each other with the sample holder placed between them. The light from the blue light passes through the sample in the plastic vial and the fluorescent light emitted from the sample (520–560 nm) was received by the camera placed beneath the sample holder. The fluorescent light emitted by the sample was filtered using a green optical filter before being received by the camera to improve the signal to noise ratio. The user can either record a video for 30 min or take snapshots of the illuminated sample at intervals of 10 min o for 30 min using the GUI. The python code was programmed to turn on the blue light in 10 min interval to avoid the photo bleaching of the fluorescent dye. A mini hot air blower was arranged to blow hot air at 100 °C to the top side of the PCR tube to prevent the condensation of the sample and improve the efficiency of fluorescent excitation. The schematic of the experimental setup is depicted in Figure 1a. Figure 1b depicts the photographs of the LAMP experiments on various samples. Figure 1b is brightness enhanced for better visibility in the manuscript.

### 2.4. Experimental

PCR tube with 25-µL sample was loaded into the sample holder heated to 65 °C. Image capture of illuminated samples was initiated by pressing ‘Snapshots’ button in GUI. Images were captured at 0, 10, 20 and 30 min of the LAMP reaction and were subsequently saved on the single board computer for data analysis and visual inspection. Each of these images were converted into RGB format and the sum of green component of each pixel of the image was automatically calculated by the python code. This sum of green component of all the pixels of the image was considered as the intensity of fluorescence of corresponding image. The fluorescent intensity value thus obtained for the image taken at 0 min was considered as the background noise and was subsequently subtracted from the fluorescent intensity values obtained for images taken at 10, 20, and 30th min for offset correction. The offset corrected values of fluorescent intensities were automatically plotted by the algorithm.

## 3. Results and Discussion

### 3.1. SARS-CoV-2 Specific LAMP Assay Using Conventional Thermocycler

**Figure 2:**
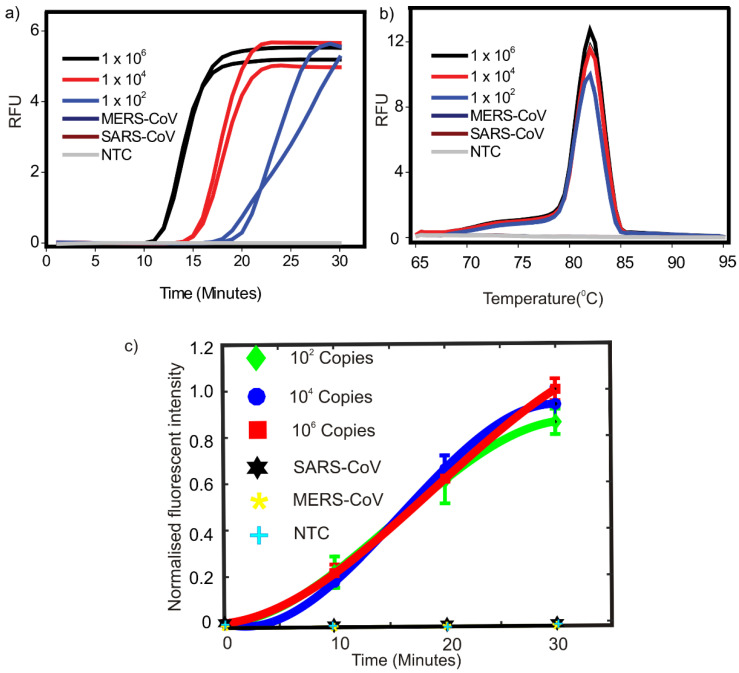
(**a**) Amplification curves obtained for LAMP reactions carried out on conventional qPCR machine. (**b**) Corresponding melt peaks. For MERS-CoV and SARS-CoV controls see Section 2.1 for details. NTC = no-template control. (**c**) Variation of normalised fluorescent intensity of various samples with respect to time on performing the LAMP.

Three different known starting copy numbers (C. Ns), 1 × 10^2^, 1 × 10^4^, and 1 × 10^6^ of vRNA were tested. A no template control (NTC) reaction was also included. As shown in Figure 2a, our LAMP assay was able to successfully detect as low as 100 starting copies of extracted vRNA. We have previously demonstrated that the LAMP primers used in this report can potentially detect as low as 10 copies of synthetic target in the presence of 1 mg/mL bovine serum albumin [18] Melt curve analysis also showed sharp peaks around 82 °C for all the concentrations (Figure 2b). In order to demonstrate the specificity of our LAMP assay in targeting SARS-CoV-2 specific sequences compared to two closely related coronaviruses, SARS-CoV and MERS-CoV controls were also included. SARS-CoV and MERS-CoV controls used 1 × 10^6^ starting copies of corresponding virus specific sequences. As can be seen in Figure 2a,b, no amplification was observed in any of the controls.

### 3.2. Performance of Portable Device for LAMP Based Detection of SARS-CoV-2

**Figure 3:**
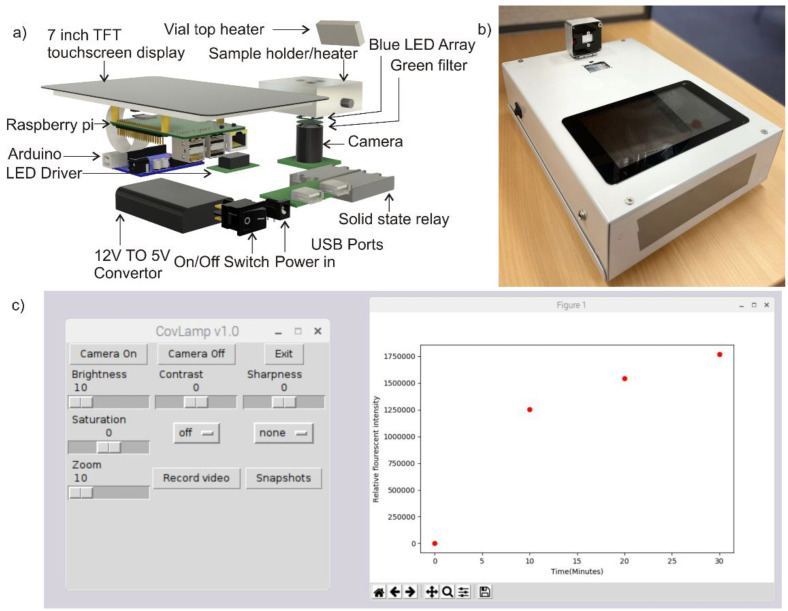
(**a**) Exploded view of the portable device. (**b**) Photograph of the portable device. (**c**) Screenshot of the graphical user interface.

For device-based LAMP reaction, three different initial extracted vRNA C. Ns 1 × 10^2^, 1 × 10^4^, 1 × 10^6^ as well as NTC and SARS-CoV and MERS-CoV controls were used. There was a clear increase in fluorescent signal intensity with respect to time for all the test samples while there was no significant visible increase in fluorescent intensity for any of the control reactions. The fluorescent intensity change was distinguishable by the naked eye by looking at the photographs taken. Images of the fluorescence emitted from the sample after every 10 min interval for samples with various initial template copy number and with negative controls are depicted in Figure 1b. The images were processed with ImageJ software to quantitatively evaluate the variation in fluorescent intensity with respect to time. The numerical equivalent values of the fluorescence were offset corrected and normalized using the equation [10,19,20,21].

(1) $$I_{s}^{*}=\left(I_{s}-I_{s0}\right)/I_{max}$$

where $I_{s}$ is the fluorescent intensity of a sample measured at a given cycle, $I_{s0}$ is the fluorescent intensity of that sample at the beginning of thermal cycling and $I_{max}$ is the maximum fluorescent intensity recorded among all the samples in the experiment. The variation of normalised fluorescent intensity of various samples with respect to time is shown in Figure 2c. Viral load in patients even at prodromal stage has been estimated to be in the range of 1 × 10^5^ copies per oro- or nasopharyngeal swab specimen [22]. Therefore, this was considered an appropriate benchmark for minimum detectable copy number. Our device was able to detect visible fluorescence signals in concentrations as low as 1 × 10^2^ within 10–20 min (Figure 1b) indicating our device can efficiently detect 1000-fold lower concentrations of extracted vRNA compared to the viral RNA copies routinely found in swab samples. Moreover, we also demonstrated the specificity of our assay. Only reactions containing SARS-CoV-2 RNA showed a clear increase in fluorescence while no amplification as indicated by increase in fluorescence signal was observed using non-specific template (SARS-CoV and MERS-CoV controls) as well as in the absence of template (NTC). One of the significant features of our assay is that it targets highly conserved RdRp region in SARS-CoV-2 genome. Our previous in silico analysis showed that the region targeted in our LAMP assay is not only conserved across various isolates and variants, but also it is highly specific for SARS-CoV-2. Our LAMP primers did not show any significant similarity with the genomes of any of the six closely related human coronaviruses [18]. The experimental setup shown in Figure 1a is subsequently organized into a portable device for user friendly operation. The difference between the experimental setup and the portable device model is that a copper block embedded with cartridge heater replaces the hot air blower to avoid condensation of the sample on the top side of the plastic vial and the illumination of the sample is done from the bottom. The detailed exploded view and appearance of the portable device is depicted in Figure 3a. Figure 3b illustrates the physical appearance of the device. Figure 3c is the screenshot of the GUI.

## 4. Conclusions

We have demonstrated the applicability of a proof-of-concept device for LAMP based detection of clinically relevant concentrations of SARS-CoV-2 within 10 to 20 min. The device proposed in this study utilizes a raspberry single-board computer, Atmega 328 microcontroller (Arduino), Raspberry pi camera, and other simple basic electronic components in its design. The total cost of the components of proposed device may be below A$150. The cost can be further reduced to below A$100 by using Raspberry pi zero single-board computers and further optimization of the design. One other salient feature of the proposed device is that it can be easily assembled by anyone with a fundamental level of expertise in electronics using locally available components thus potentially helping to circumvent the mass scale production and supply issues. The proposed handheld device can be easily deployed in places such as airports or hospital emergencies where rapid detection and subsequent quarantine is highly recommended. Its application in POC settings will not only facilitate in rapid clinical management decisions but will also reduce the burden of centralized testing laboratories. The device opens an avenue for high throughput, low cost, and rapid detection of SARS-CoV-2 in the present pandemic scenario. Moreover, the proposed handheld LAMP device can be easily adapted for the LAMP based rapid detection of many other diseases. Furthermore, droplet-based microfluidics would allow for faster two-phase flow heat transfer [23], droplet manipulation [24] and more effective LAMP reaction. In the near future, flexible systems allow for the integration temperature sensing and heating on low-cost substrate such as paper [25,26], the concept described in this paper can be further simplified to lower the assay cost.

## Figures and Tables

**Table 1:** Sequences of templates and primers.

	Sequence
Cor-RdRp-F3	GCTCGCAAACATACAACGT
Cor-RdRp-B3	GTTACCATCAGTAGATAAAAGTGCA
Cor-RdRp-FIP	CGCCACACATGACCATTTCACTCAATTTTGTTGTAGCTTGTCACACCGT
Cor-RdRp-BIP	AGGTGGAACCTCATCAGGAGATGTTTTAACATTGGCCGTGACAGC
Cor-RdRp-LF	CTTGAGCACACTCATTAGCTAATC
Cor-RdRp-LB	CCACAACTGCTTATGCTAATAGTGT
